# Cardiovascular magnetic resonance in pericardial diseases

**DOI:** 10.1186/1532-429X-11-14

**Published:** 2009-05-04

**Authors:** Jan Bogaert, Marco Francone

**Affiliations:** 1 Department of Radiology, UZ Leuven, B-3000 Leuven, Belgium; 2 Department of Radiology, University of La Sapienza, Rome, Italy

## Abstract

The pericardium and pericardial diseases in particular have received, in contrast to other topics in the field of cardiology, relatively limited interest. Today, despite improved knowledge of pathophysiology of pericardial diseases and the availability of a wide spectrum of diagnostic tools, the diagnostic challenge remains. Not only the clinical presentation may be atypical, mimicking other cardiac, pulmonary or pleural diseases; in developed countries a shift for instance in the epidemiology of constrictive pericarditis has been noted. Accurate decision making is crucial taking into account the significant morbidity and mortality caused by complicated pericardial diseases, and the potential benefit of therapeutic interventions. Imaging herein has an important role, and cardiovascular magnetic resonance (CMR) is definitely one of the most versatile modalities to study the pericardium. It fuses excellent anatomic detail and tissue characterization with accurate evaluation of cardiac function and assessment of the haemodynamic consequences of pericardial constraint on cardiac filling. This review focuses on the current state of knowledge how CMR can be used to study the most common pericardial diseases.

## Pericardial anatomy and physiology

Although the normal pericardium is a thin, avascular, relatively inelastic, flask-shaped sac enveloping the heart, this structure is an important determinant of cardiac filling [1-3]. In most cases, evaluation of pericardial diseases extends beyond morphologic assessment, and the diagnostic challenge is to determine the impact of abnormal pericardium on cardiac filling. Anatomically, the pericardium is composed of two layers, an inner serous membrane and an outer fibrocollagenous layer [3]. The inner serosal layer, termed the visceral pericardium, is closely attached to the epicardial surface of the heart and covers a subepicardial layer of conjunctive tissue containing fat and coronary vessels. The serosal layer reflects back on itself to become the inner lining of the outer fibrous layer. Together, these layers form the parietal pericardium. The pericardium contains two major pericardial sinuses which are composed of different recesses [4]. The transverse sinus is the connection between the two tubes of pericardium that envelop the great vessels. The aorta and pulmonary artery are enclosed in one anterosuperior tube, and the vena cava and pulmonary veins are enclosed in a more posterior tube. The oblique sinus lies behind the left atrium so that the posterior wall of the left atrium is actually separated from the pericardial space. This explains why a posterior pericardial effusion is seen behind the left atrium only when it is very large. The pericardial virtual cavity normally contains between 10 and 50 ml of an ultrafiltrate of plasma [5]. The fluid is produced by the visceral pericardium and drainage of the cavity is toward both the thoracic duct and the right lymphatic duct.

The pericardium holds the heart within the anterior mediastinum, protecting it form adjacent organs. Its relative inelasticity provides constraint during diastolic filling which limits chamber dilation, particularly of the thin-walled right atrium and ventricle [2]. While acute pericardial dilation is limited because of its exponential pressure-volume relation, chronic pericardial stress, e.g. slowly accumulating pericardial effusion or left ventricular remodeling, in contrast, may occur without pericardial constriction [1,6,7]. As the pericardium slowly dilates, the pericardial pressure-volume relation shifts to the right, explaining why chronic pericardial effusion may become quite large without compressing the cardiac chambers [8]. The pericardium, moreover, equalizes compliance between right and thicker walled left ventricle, and produces interdependence of filling between ventricles, a phenomenon also called "ventricular coupling" (see constrictive pericarditis). Though, normally not physiologically important, ventricular coupling is exaggerated and a key diagnostic feature when intrapericardial pressure is increased (as in cardiac tamponade) or when the pericardial cavity is fixed (as in constrictive pericarditis) [2,6-10]. Finally, the pericardium acts also as a physiological intermediate between the pleural space and heart chambers, and the respiratory changes in intrathoracic pressure are directly transmitted to the subatmospheric pericardial cavity and subsequently to the cardiac chambers. Because of its thinner wall, this interaction is more pronounced on the right than on the left ventricle.

## CMR techniques

Comprehensive pericardial imaging should provide information on morphologic characteristics of the pericardium and cardiac structures, and assess the impact of pericardial diseases on cardiac function, in particular cardiac filling. Though CMR is traditionally considered together with computed tomography (CT), as the preferred imaging modality to morphologically visualize the pericardium and pericardial space, CMR can substantially aid in clarifying the intricate relation between pericardial constraint and cardiac filling. Black-blood T1-weighted spin-echo CMR, using a fast, segmented sequence, is the best approach to visualize, the heart, pericardium and mediastinum [11,12]. Use of a small field of view and a saturation block positioned on the frontal chest wall may hereby improve pericardial visualization. Scanning in two perpendicular oriented planes through the heart guarantees optimal depiction of the entire pericardium, for instance, using a set of axial views combined with coronal or cardiac short-axis views. T2-weighted spin-echo CMR, preferably using a short-tau inversion-recovery (STIR) sequence (also called "triple-inversion" spin-echo), highly useful to depict myocardial edema [13,14], can also be applied to detect pericardial fluid and/or edema of the pericardial layers in patients with inflammatory pericarditis. Use of paramagnetic contrast agents can be recommended in case of pericardial masses, inflammatory pericarditis, to depict concomitant myocardial pathology (e.g. myocarditis) and may be useful to better differentiate between inflammatory and constrictive forms of pericarditis. Either T1-weighted spin-echo CMR or inversion-recovery gradient-echo CMR with late enhancement (LGE CMR) is fit for post-contrast imaging [15-17].

Cine CMR, using balanced steady-state free precession (SSFP) gradient-echo sequences, is today the reference technique to quantify global and regional cardiac systolic function, and is of interest for example to rule out underlying RV or LV dysfunction in patients clinically suspected of constrictive pericarditis. Moreover, the high spatial and temporal resolution of cine CMR can be applied to exploit new applications such as the assessment of pericardial mobility, which can help in depicting a rigid pericardium in patients with a non or minimally thickened constrictive pericarditis. The availability of new real-time cine sequences enabled to study dynamic, fast-changing, physiologic events such as ventricular coupling (see below)[18]. CMR tagging techniques may be of use to detect fibrotic adhesion of pericardial layers or to diagnose myocardial involvement in constrictive pericarditis [19]. Assessment of diastolic heart function, though usually obtained with Doppler-echocardiography, can be achieved with velocity-encoded or phase-contrast CMR technique too [11,20,21]. Analysis of pulmonary and/or systemic venous pattern in combination with cardiac inflow patterns through the atrioventricular valves can yield findings that are classic for restrictive cardiac filling.

To conclude, improvements in CMR technology have shifted the way this technique can be applied to study the pericardium from basically a static morphologic assessment toward an integrated dynamic morphological-functional approach, raising the hope to improve diagnostic testing to the often complex and intriguing group of pericardial disorders (Appendix 1).

## Normal pericardium

**Figure 1:**
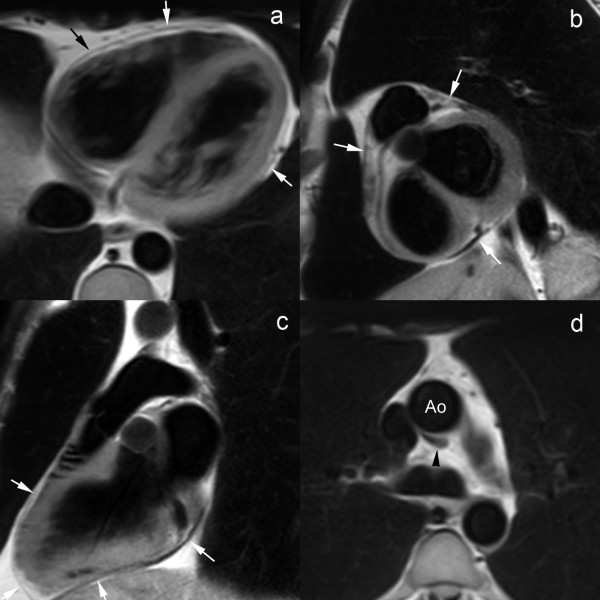
**Normal pericardium on T1-weighted fast spin-echo CMR in axial direction**. (a), short-axis (b), and vertical long-axis (c), and axial view of superior aortic pericardial recess (d). The normal pericardium is visible as a thin curvilinear structure (arrows) with low signal intensity surrounded by the bright epicardial and mediastinal fat (a-c). Pericardial visualization is often hampered along the free wall of the left ventricle (b). The superior aortic recess, part of the transverse sinus is visible posterior to the ascending aorta, visible as a hypo-intense crescent structure (black arrowhead) (d).

Normal pericardium is visible on spin-echo CMR as a thin, smooth, low-intensity curvilinear structure surrounded by high-intensity mediastinal and epicardial fat, or medium-intensity myocardium (Fig. 1) [22,23]. The low signal of adjacent lung parenchyma and paucity of surrounding fat, may hamper the pericardial visualization over the free wall of the left ventricle [22]. The transverse pericardial sinus, preaortic and retroaortic recesses can be identified in the majority of patients [23,24]. Especially the superior pericardial recesses should not be mistaken for a focal aortic dissection or enlarged lymph nodes (Fig. 1d) [4,25,26]. On b-SSFP cine CMR, the pericardial layers have a low signal intensity while pericardial fluid has a high signal intensity. On CMR, normal pericardium measures 1.2 mm in diastole to 1.7 mm in systole, which are larger than those found in anatomical studies of the heart, ie, 0.4–1 mm [27-29]. The overestimation of pericardial thickness on CMR is due to motion of pericardial layers, lack of sufficient spatial resolution, and chemical shift artifacts at the fat-fluid interface on cine CMR.

## Congenital pericardial anomalies

### Pericardial Cyst

**Figure 2:**
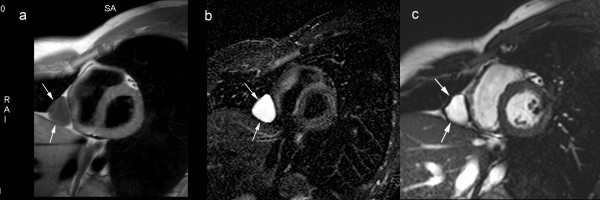
**Congenital pericardial cyst**. **T1-weighted spin-echo CMR**. (a) T2-weighted short-tau inversion-recovery spin-echo CMR (b), and cine CMR (c) in cardiac short-axis. The pericardial cyst is visible as a well-delineated, homogeneous soft-tissue structure (arrows) along the right paracardiac border implanted on a normal pericardium. The cyst content typically has signal characteristics of water on CMR.

Pericardial cysts are congenital encapsulated cysts implanted on the pericardium that are not connected with the pericardial cavity. They typically occur in the cardiophrenic sulcus (90%), most often right-sided (70%). On chest radiography, they present as a well-defined outpouching on the lateral heart border. On CMR, they appear as a well-defined homogeneous paracardiac structure, having the signal characteristics of water, implanted on the pericardium (Fig. 2)[27]. Pericardial cysts are usually asymptomatic, though rarely they may become symptomatic when compressing other cardiac structures. Pericardial cysts should be differentiated from encapsulated pericardial effusions, and other cystic structures such as bronchogenic cysts and thymic cysts.

### Pericardial Defect

Congenital pericardial agenesis is an uncommon entity, and is considered to result from an abnormal embryonic development that may be secondary to abnormalities in the vascular supply of the pericardium. Pericardial defects occur in a spectrum ranging from a small defect to total absence of the pericardium. Partial defects (large more common than small partial defects) are far more common than total defects [27,30]. It may be associated, in at least one-third of cases, with other malformations, particularly malformations of the heart (tetralogy of Fallot, atrial septal defect, patent ductus arteriosus) or other types of abnormalities (bronchogenic cyst or hiatus hernia) [31,32]. Cardiac structures or portions of the lung can herniate through the defect. The clinical presentation is variable. Patients are often asymptomatic, and the disease may be detected on routine chest radiograph as an abnormal left cardiac contour [33]. Symptoms occur when cardiac structures are transiently entrapped or incarcerated in the defect. Herniation of the left atrial appendage through a small defect may lead to infarction of the appendage or the left coronary artery might be compressed leading to ischaemia especially during exercise [30].

The diagnosis of a pericardial defect is not always straightforward on CMR, since in normal conditions the pericardium over the lateral side of the left ventricle, corresponding to the most frequent location of pericardial defects, is usually not well depicted because of a paucity of surrounding fat [34]. So, the diagnosis usually relies on other signs such as an abnormal location of cardiac structures with excessive levorotation or cardiac indentation at the location of the defect [35,36]. Since herniation is often intermittent in time, positional changes such as to positioning the patient in left lateral decubitus can be helpful in diagnosing pericardial defects. Functional examination may be helpful in establishing the diagnosis of congenital pericardial defect. Whereas the normal cardiac apex is essentially stationary in the chest during the cardiac cycle, excessive mobility may be indicative of a pericardial defect [37].

### Pericardial Diverticulum

Pericardial diverticulum is an exceedingly rare condition that can be congenital or acquired. It corresponds to a herniation through a defect in the parietal pericardium that communicates with the pericardial cavity [27]. Congenital diverticula result from a failure in the fusion of one of the mesenchymal lacunae that normally concur to form the pericardial sac. They typically occur in the cardiophrenic angles and have the tendency to change in size over time. CMR is helpful in reaching a preoperative diagnosis. Although it resembles a pericardial cyst, the diagnosis of a diverticulum should be suspected when a complete wall cannot be identified in all parts of the lesion [38].

## Acquired pericardial diseases

### Pericardial Effusion

**Figure 3:**
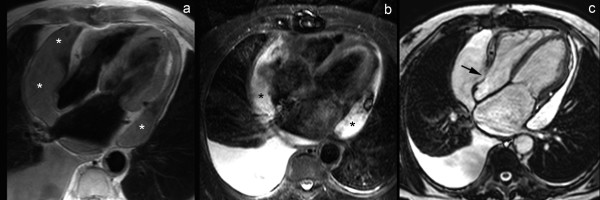
**Moderate pericardial effusion**. **T1-weighted spin-echo CMR**. (a), T2-weighted short-tau inversion-recovery spin-echo CMR (b), and cine CMR (c) in axial image plane. The pericardial effusion (arrowheads), having an inhomogeneous spread, is most pronounced over the right atrium and left ventricle. Systolic collapse of the right atrial wall during systole (arrow)(c). The fluid signal intensity, especially on T2-weighted short-tau inversion-recovery spin-echo CMR, has an inhomogeneous appearance (b). Moderate right-sighted pleural effusion. The collapse of right atrial and right ventricular wall during the cardiac cycle can be well appreciated on cine CMR.

**Figure 4:**
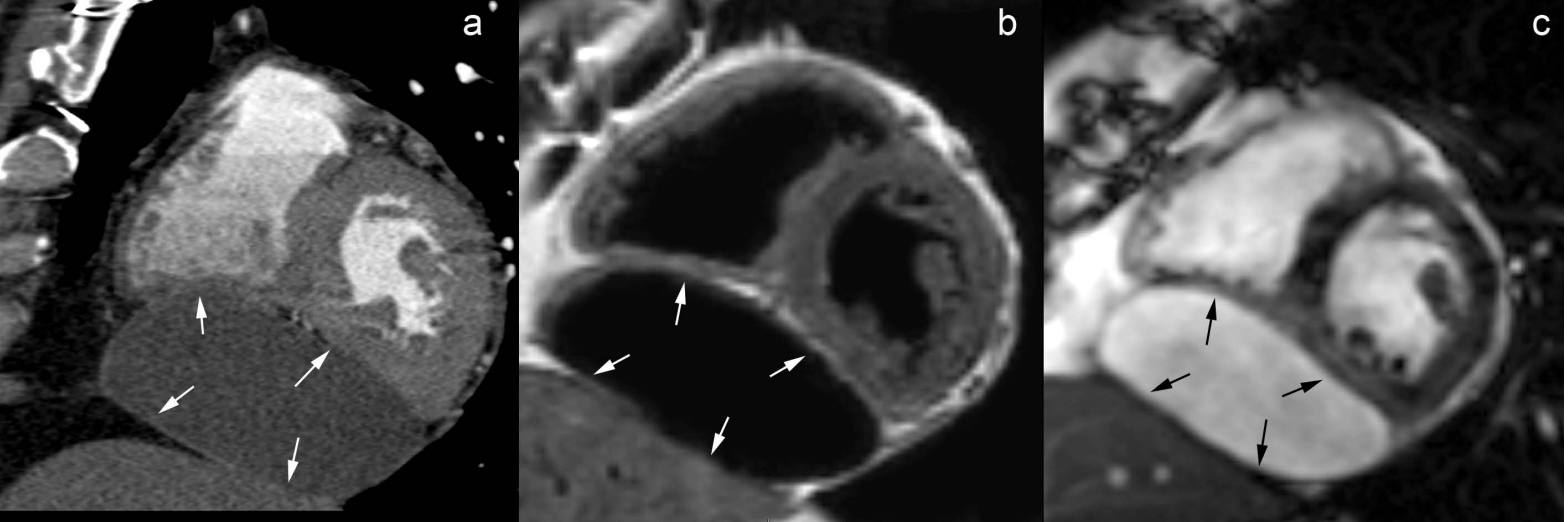
**Loculated pericardial effusion with cardiac compression**. Computed tomography (a), T1-weighted spin-echo CMR (b) and balanced SSFP CMR (c). All images shown in cardiac short-axis. Large oval-shaped pericardial effusion (arrows), inferiorly located to the heart. Note the compression on RV and LV.

Abnormal fluid accumulation may be seen in heart failure, renal insufficiency, infection (bacterial, viral, tuberculous), neoplasm (carcinoma of lung, breast, lymphoma), trauma, and myocardial infarction [39]. Imaging is often required to confirm the presence, severity and extent of fluid; to characterize the nature of fluid; to rule out pericardial inflammation; to determine the haemodynamic impact on the heart; and to guide pericardiocentesis. For this purpose, echocardiography is the standard. However, image quality and interpretation may be hampered by acoustic window, and in obese patients or in those with obstructive lung disease, necessitating additional imaging, such as CT or CMR. With exception of transesophageal echocardiography, this technique is generally unreliable for assessment of pericardial thickness, and may be of limited value in detecting loculated effusions [2,11,12]. Because the pericardial sac can be easily and completely visualized on CMR, this technique is superior to detect the distribution and amount of fluid accumulation than with echocardiography [40]. Although CMR can detect pericardial effusions as small as 30 ml, a clear-cut relationship between the measured width of the pericardial space and total fluid volume cannot be established because pericardial fluid accumulation is often not homogeneously spread (Fig. 3). Due to the gravitational dependency, focal fluid accumulaton posterolateral to the left ventricle and along the inferolateral wall of the RV are not infrequent (Fig. 4)[23]. Another common location is the superior pericardial recess. In general, a pericardial width greater than 4 mm should be regarded as abnormal. Moderate effusions (between 100 and 500 ml of fluid) are associated with a greater than 5 mm pericardial space anterior to the right ventricle [22,28]. Similar to ventricular volumetric quantification, a multislice approach can be used to quantify the precise amount of pericardial fluid.

Because the stretching capacity of the pericardial layers is limited, acute accumulation of pericardial contents (e.g., fluid, blood, air) can lead to cardiac chamber compression with decreased cardiac filling and subsequently impaired stroke volume, a phenomenon called "cardiac tamponade" [40]. The symptomatology can be dramatic, potentially lethal within minutes after onset. Symptoms are linked to the increase in pericardial pressure determined by the absolute volume of fluid, rate of fluid accumulation, and the physical characteristics of the pericardium (degree of stretching)(Fig. 4). The diagnosis of pericardial tamponade is a clinical one that is usually confirmed by echocardiography. Hallmarks of cardiac tamponade are the diastolic inversion or collapse of the RV free wall indicating intrapericardial pressure exceeding interventricular pressure; right atrial compression during early systole; exaggerated respiratory variation in cardiac inflow; and abnormal flow patterns in the caval veins [41]. Though, CMR has a limited role in the diagnosis of acute cardiac tamponade; chronic, less severe, forms can be encountered in patients with pericardial effusion on CMR (Fig. 3c) [see Additional file 1]. Care should be taken to look for features of constriction, which can occur transiently in the resolution phase, after pericardiocentesis or with organized effusions (see below effusive-constrictive pericarditis)[2].

Analysis of CMR signals enables, at least to some extent, fluid characterization. Transudates typically have a low signal intensity on T1-weighted spin-echo CMR, and a high signal intensity on T2-weighted sequences. Exudates, having a high protein and cell content, increase T1-relaxation (higher signal intensity) and shorten T2-relaxation (lower signal intensity)(Figs. 3, 4). However, because of motion artifacts, pericardial fluid characterization is not always feasible. In particular non-linear motion of pericardial fluid during cardiac motion may falsely cause high signal on T1-weighted spin-echo CMR within the effusion even in the presence of a simple transudate. Moreover characterization of small effusions may be often difficult. The signal intensity of a hemorrhagic pericardial effusion is dependent on the duration of the disease (see pericardial hematoma). With balanced SSFP-gradient-echo techniques, often a better characterization of the pericardial fluid content can be achieved such as the visualization of fibrinous strands or presence of coagulated blood. Other abnormalities helpful in the diagnosis are depiction of thickened pericardial blades with assessment of pericardial inflammation [11,17].

### Pericardial Inflammation

Inflammation of the pericardium (*inflammatory pericarditis*) presents in many clinical settings and has a wide range of causes [2]. The acute presentation is an important differential in the assessment of acute chest pain, but pericarditis can also present in subacute or chronic forms. The symptoms are mainly related to the severity of pericardial inflammation. Although the true incidence and prevalence of pericarditis are difficult to measure, a prevalence of 1% in autopsy studies suggests that pericarditis may be subclinical [2]. In up to 30% of patients no cause of pericarditis can be defined (idiopathic). Infections (viral/bacterial/tuberculosis/fungal) are common causes of acute pericarditis. It can be noted that organisms responsible for myocarditis are commonly implicated. In developed countries, tuberculosis has become less common, but should still be considered in immunocompromised hosts [1,2,42]. Radiation therapy for treatment of mediastinal tumors and breast cancer is an increasingly important cause of pericarditis and pericardial constriction. Pericarditis can be a manifestation of various systemic diseases (e.g. rheumatoid arthritis, systemic lupus erythematosus, scleroderma); can be secondary to primary or metastatic pericardial disease, and can be found in patients with uremia or following an acute myocardial infarction. The latter occurs early postinfarction and is typically found in transmural infarctions. This condition should be differentiated from late postinfarction pericarditis (Dressler's syndrome). While acute post-infarction pericarditis, also called "epistenocardic pericarditis", has a close temporal relation with the acute event due to the pericardial spread of infarct-related inflammation, Dressler's syndrome has an autoimmune etiology without a close temporal relation with myocardial infarction. Finally, direct or indirect trauma can cause traumatic pericarditis.

In the acute phase, inflammation of the pericardial layers is characterized by formation of young, highly vascularized granulation tissue with fibrin deposition [17]. Usually, a variable amount of pericardial fluid is present, and the fibrin deposition may lead to a fibrinous adhesion of the pericardial layers. Chronic inflammation is characterized by a progressive sclerosing pericarditis with fibroblasts, collagen and a lesser amount of fibrin deposition [18]. This may progress towards an end-stage, chronic fibrosing pericarditis with fibroblasts and collagen. The main feature of this end-stage is a stiff pericardium with constriction of the heart (*constrictive pericarditis*).

**Figure 5:**
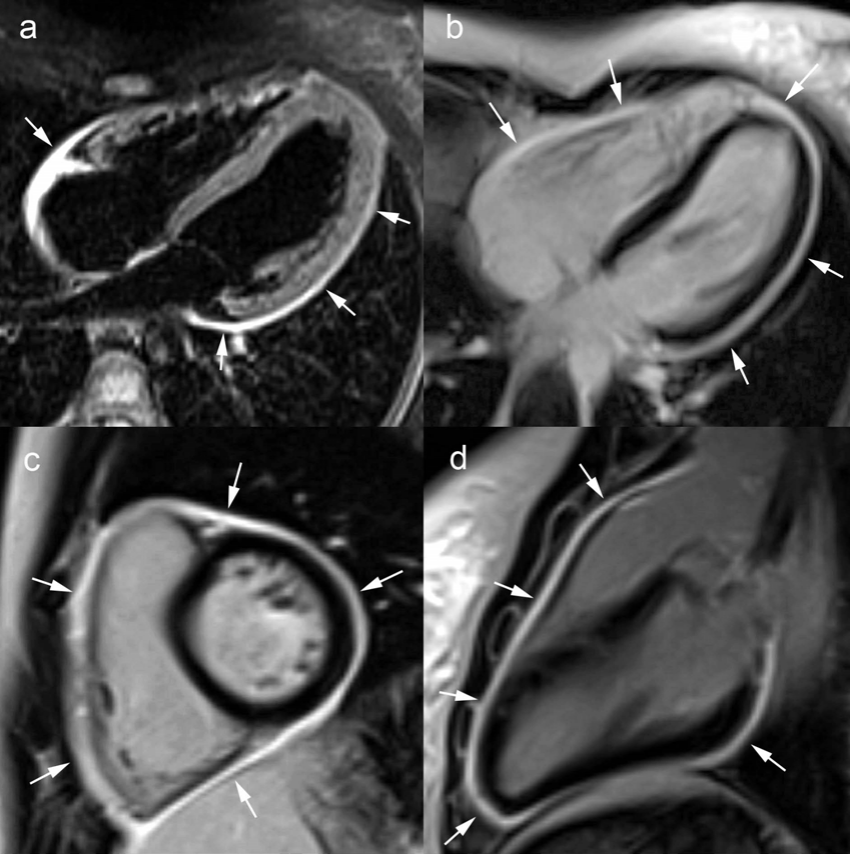
**Acute viral inflammatory pericarditis in 15-year-old girl**. T2-weighted short-tau inversion recovery spin-echo CMR (a), LGE CMR in horizontal long-axis (b), short-axis (c) and vertical long-axis plane (d). Diffuse hyperintense appearance of the pericardium on T2-weighted short-tau inversion recovery spin-echo CMR (arrows)(a). Strong, homogeneous enhancement of the entire pericardium following gadolinium administration (arrows)(b, c, d).

**Figure 6:**
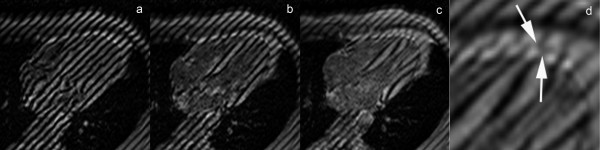
**CMR tagging using the SPAMM (spatial modulation of magnetization) technique in a healthy volunteer in axial plane**. (a, b, c) and enlarged view at end systole (d). The tags are oriented in long-axis direction, and positioned on the heart at end diastole. Because of shear motion between visceral and parietal layer, the continuity of the tags gets lost during cardiac contraction (arrows)(d).

Detection of inflammatory pericarditis has become less challenging with the availability of modern CMR techniques [11,12,19]. Both thickening of pericardial layers, and depiction of associated pericardial effusion can be well depicted on T1-weighted spin-echo CMR or cine CMR, while T2-weighted STIR spin-echo CMR allows visualization of edema of the inflamed pericardial layers (Fig. 5). Pericardial enhancement on gadolinium-enhanced CMR studies is an appropriate way to detect pericardial inflammation (Fig. 5)[17]. Both LGE CMR or spin-echo CMR are useful. Addition of a fat-suppression prepulse may be interesting to enhance visualization of pericardial inflammation. Other interesting imaging features are assessment of pericardial layer delineation, which might become more irregular in cases of chronic pericarditis and streaky enhancement of the surrounding fat and enhancement of adjacent myocardial tissue, indicating associated epicarditis or myocarditis, respectively. Today, CMR is considered the non-invasive standard to depict myocarditis [43,44]. In a recent study, Yelgec and colleagues reported pericardial enhancement in 9 and pericardial effusion in 6 patients in a group of 20 patients studied with CMR for clinical suspicion of acute myocarditis [45], indicating myopericarditis is not an uncommon presentation. Tagging CMR techniques may be of use to well depict fibrotic adhesions of pericardial layers (Fig. 6) [see Additional file 2] [19].

### Constrictive Pericarditis

Chronic fibrosing pericarditis is characterized by a thickened, fibrotic and/or calcified pericardium, not infrequently constricting the heart and impairing cardiac filling ("constrictive" pericarditis) [46]. Patients with pericardial constriction typically present with manifestations of elevated systemic venous pressures and low cardiac output [47]. The diagnosis should always be considered in patients presenting with predominant right heart failure symptoms [48]. The pathophysiological hallmarks of pericardial constriction, which are caused by confinement of the cardiac chambers by the rigid, fixed pericardial volume, are equalization of end-diastolic pressures in all 4 cardiac chambers, and increased ventricular coupling which is strongly influenced by respiration [1,2].

Pericardial constriction is most commonly idiopathic but can be the end result of any cause of inflammatory pericarditis. Tuberculosis has become less frequent, while cardiac surgery and radiation-induced pericarditis have become also important [2]. In non-tuberculous pericarditis pericardial thickening and calcification may be less prominent. In radiation therapy, systolic dysfunction may accompany constriction and is a marker of poor prognosis after pericardiectomy. Pathology typically shows an avascular collagen-rich, fibrotic tissue, often containing areas of calcification. Moreover, in extensive cases the fibrosing process may be adherent or even involve the myocardium.

Today, despite a wide arsenal diagnostic tools, the diagnosis of constrictive pericarditis often remains a challenge, and the diagnostic approach taken should be individualized for each patient. First, all other causes of (right) heart failure (e.g. pulmonary hypertension, LV/RV infarction) should be excluded. Second, it should be determined whether the pericardium is causing constriction hereby impeding cardiac filling, and thirdly whether the patient will benefit of a pericardial stripping. One of the major dilemmas, is that diseases with decreased myocardial compliance, such as restrictive cardiomyopathy, are also characterized by impaired cardiac filling, and may present in a very similar way. Distinction between the two entities is crucial, because constrictive pericarditis patients are potentially successfully treated by early pericardiectomy whilst for restrictive cardiomyopathy medical treatment is recommended [46-50]. Several other factors may hamper straightforward diagnosis of constrictive pericarditis. The morphologic pericardial abnormalities classically found in constrictive pericarditis may be not impressive, be absent or have an atypical presentation. Moreover, increased pericardial thickness does not necessarily imply pericardial constriction, and vice versa [2]. Finally, for instance in patients with a history of radiation therapy, restrictive cardiomyopathy and constrictive pericarditis may simultaneously occur. This makes the diagnosis of constrictive pericarditis, especially in atypical or complex cases, often challenging. Since no one method is completely reliable, information from more than one imaging technique should be considered to provide an assessment of anatomical and physiological function [48].

#### Morphologic Abnormalities

**Figure 7:**
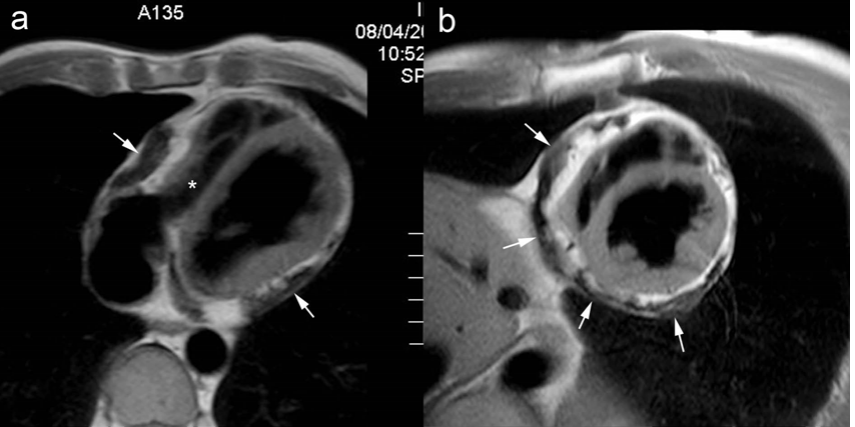
**Classic presentation of constrictive pericarditis on T1-weighted fast spin-echo CMR, axial view (a), and short-axis view (b)**. Extensive pericardial thickening is found on the laterobasal and inferior part of both right and left ventricle and atrioventricular grooves (arrows). The thickened areas are irregularly defined, and are compressing the adjacent cardiac cavities, mainly on the right (*).

**Figure 8:**
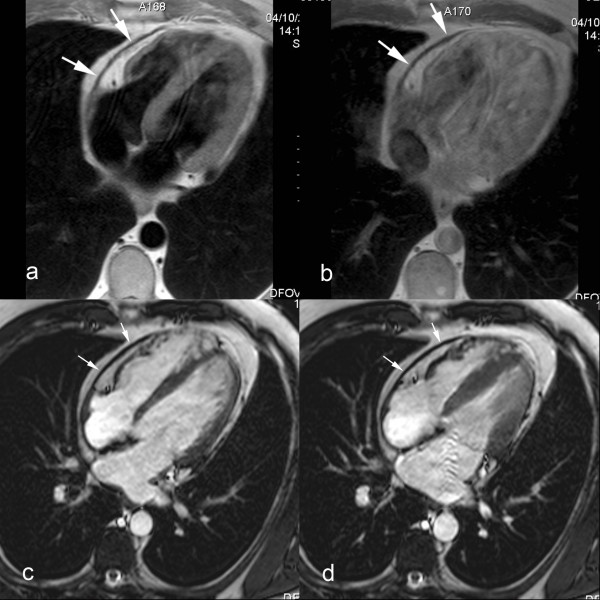
**Calcified constrictive pericarditis, axial T1-weighted spin-echo CMR before (a) and after (b) administration of gadolinium-chelates, cine CMR at end diastole (c) and end systole (d) in the same imaging plane**. A mildly thickened, hypointense appearing pericardium is visible over the right heart (arrows). No enhancement of the thickened pericardium after gadolinium administration (b). The thickened part of the pericardium has a rigid, immobile appearance, while the underlying myocardium exhibits a normal contraction pattern. The dynamic viewing mode allows a better appreciation of the differences in motion between the rigid pericardium and underlying myocardium.

The typical morphological presentation of constrictive pericarditis is a more or less generalized thickening of the pericardium. This thickening is usually most pronounced over the right heart side (right ventricle and anterior atrioventricular groove), and the pericardial delineation is often irregular (Fig. 7)[51]. The underlying cardiac cavities may be constricted by the abnormal pericardium, having a flattened or tubular-shaped appearance. Indirectly, as a result of the increased cardiac filling pressures, unilateral or bilateral atrial enlargement, dilatation of caval and hepatic veins, pleural effusion, ascites are encountered. Useful criteria to assess pericardial thickness by CMR are a) pericardial thickness 2 mm or less: normal, b) pericardial thickness greater than 4 mm: suggestive of pericardial constriction in patients with the appropriate clinical presentation, c) pericardial thickness greater than 5–6 mm: high specificity for constriction [52-54]. The thickened fibrotic and/or calcified pericardium has a low signal not only on T1- and T2-weighted spin-echo CMR but also on cine imaging (Fig. 8). In end-stage chronic fibrosing forms of constrictive pericarditis there is no enhancement after gadolinium (Fig. 8) [see Additional file 3]. [17]. Pericardial enhancement is suggestive of residual inflammation. Differentiation between pericardial thickening and effusion is usually straightforward using a comprehensive CMR approach. Today, CT is the most appropriate technique to depict, even minute amounts of pericardial calcium, whereas significant deposits may be missed by CMR.

**Figure 9:**
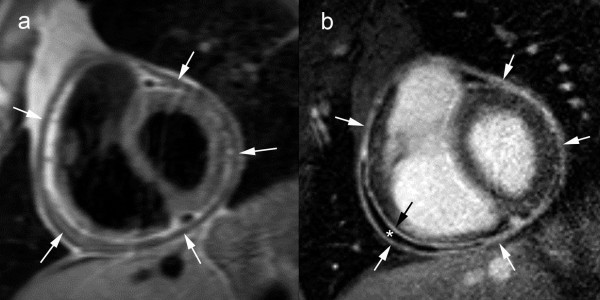
**Inflammatory-constrictive form of constrictive pericarditis**. Short-axis T1-weighted spin-echo CMR (a) and LGE CMR (b). Diffusely thickened pericardium, with strong enhancement of the pericardial layers after gadolinium administration (arrows). Presence of a small pericardial effusion (*) along the inferolateral side of the right ventricle. Real-time CMR in cardiac short-axis during free breathing shows increased ventricular coupling with inspiratory septal inversion and increased right-sided septal motion at onset of expiration. At surgery, a heavily thickened, inflamed and constrictive pericardium was found.

**Figure 10:**
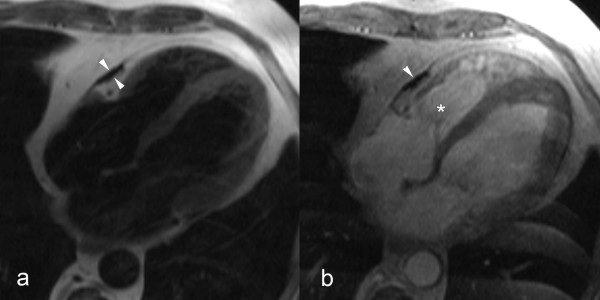
**Focal constrictive pericarditis, axial T1-weighted spin-echo CMR (a) and axial cine CMR (b)**. Focal pericardial thickening (arrowheads) at the level of the right atrioventricular groove and basal part of the free wall of the right ventricle with focal compression of right ventricular cavity (*). The very low signal intensity of the focally thickened pericardium on T1-weighted spin-echo CMR as well cine CMR should alert one to look for calcification of the pericardium.

In a considerable number of patients the classical morphologic findings are lacking. Pericardial constriction may occur in any condition where the compliance of the pericardium is decreased. *Effusive-constrictive pericarditis *is a condition where the constriction is not relieved after removal of the pericardial fluid [55-57]. This condition was found in 15 of 218 pericarditis patients presenting with cardiac tamponade [55]. It is believed the non-compliant visceral pericardium is causing the constriction. The diagnosis of effusive-constrictive pericarditis is often challenging because the morphologic abnormalities, even at visual inspection, are not impressive [57]. It is believed that effusive-constrictive pericarditis most likely represents an intermediate transition from acute pericarditis with pericardial effusion to pericardial constriction [57]. *Transient cardiac constriction *is not uncommon in patients with effusive acute idiopathic pericarditis [58,59]. Features of constriction may be detected in the phase of resolution of pericarditis, at a time when signs of activity have abated, and residual effusion is minimal or has disappeared entirely. The clinical and haemodynamical features of constriction spontaneously subside after a couple of months [56]. Most likely the inflammatory thickened pericardium has a decreased compliance causing symptoms of temporary constriction (inflammatory-constrictive form)(Fig. 9) [see Additional file 4]. Both entities underscore the variable evolution in patients with acute pericarditis, and represent an intermediate between spontaneous resolution and evolution toward constrictive pericarditis. The ability to differentiate fluid from pericardial layer, and to depict pericardial inflammation, makes CMR appealing to assess these atypical forms of constrictive pericarditis [11,15,17]. In some cases pericardial abnormalities might be very localized (*focal constrictive pericarditis*). Symptoms of constriction, will primarily depend on the location of abnormalities and degree of constriction. Focal constriction, for example, at the level of atrioventricular grooves or basal portion of the ventricles, though visually not impressive may significantly impede cardiac filling (Fig. 10) [see Additional file 5] [60,61]. This urges for a scrutinized visualization of the entire pericardium with CMR. Since cardiac constriction is caused by decrease in pericardial compliance and not by the pericardial thickness as such, taking the latter parameter as an absolute criterion may create a diagnostic dilemma in patients with haemodynamic findings of constriction without increased pericardial thickness. Up to 18% of patients with histologically proven constrictive pericarditis have a normal or near normal pericardial thickness (i.e. < 2 mm) (*non or minimally thickened constrictive pericarditis*) [62]. Moreover, in some patients physical and haemodynamic features of constriction are not apparent in their baseline state, but when rapidly fluid challenged, they will present a typical haemodynamic constrictive pericarditis pattern. This subgroup is called *occult constrictive pericarditis *[56,63], and shows the close interaction between cardiac filling status and physical properties of the surrounding pericardium.

Though beyond the scope of this paper, it should be emphasized CMR has an increasingly important role in investigating patients with restrictive cardiomyopathy [11]. Myocardial enhancement patterns on LGE CMR may be strongly indicative of specific myocardial infiltrative or storage diseases, while T2*-weighted CMR may be used to non-invasively depict iron deposition cardiomyopathy [11].

#### Functional and Haemodynamic Abnormalities

The consequences of encasement of the heart by a rigid pericardium are basically threefold, (a) dissociation between intrathoracic and intracardiac pressure, isolating the heart from normal respiratory changes in intrathoracic pressure, (b) increased ventricular coupling, (c) increased cardiac filling pressures with pressure equalization in all 4 cardiac chambers. Assessment of these effects is usually performed by echocardiography and cardiac catheterization, [1,2,46,64-67]. CMR, with the exception of intracardiac pressure measurements, may provide valuable information too, and has the intrinsic advantage that findings may be directly linked to morphologic abnormalities. Velocity-encoded CMR typically shows a restrictive filling pattern with an enhanced early filling, and decreased or absent late filling, depending on the degree of pericardial constriction and increased filling pressures. Also the venous flow patterns may show restrictive physiology with diminished or absent forward, or even reversed systolic flow, and increased early diastolic forward flow and late backflow. Constrictive pericarditis, in contrast with restrictive myocarditis, is typically characterized by a strong respiratory-related variation in cardiac filling (i.e., enhanced RV filling on inspiration, enhanced LV filling on expiration)[65,66]. Real-time velocity CMR is a potential alternative to echo-Doppler to assess the effects of respiration on cardiac filling, though ideally slice-tracking techniques are needed to compensate for through-plane motion [67].

**Figure 11:**
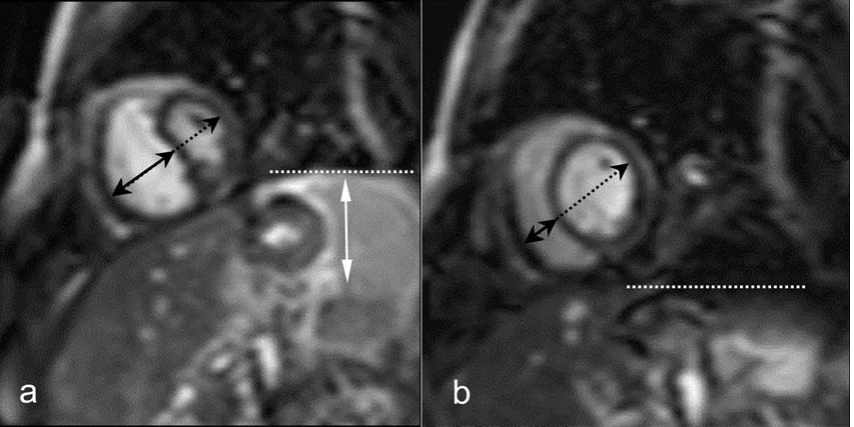
**Assessment of increased ventricular coupling using real-time cine CMR in constrictive pericarditis**. Short-axis cine CMR at onset of inspiration (a) and onset of expiration (b). Images are acquired at early ventricular filling. Septal inversion occurs at inspiration (a), with increased right-sided septal motion at expiration (b), leading to an abnormal respiratory septal shift. The horizontal dashed lines indicate the position of the left hemidiaphragm.

Since the pressure difference between the ventricles (or trans-septal pressure gradient) determines the position and configuration of the ventricular septum, this information can be used to determine the degree of ventricular coupling (or ventricular interdependence) [9,10,48,68-71]. Under normal loading conditions, due to a positive left-to-right trans-septal pressure gradient, the septum has a right-sided convex shape, and this shape is maintained during the cardiac cycle and is minimally influenced by respiration. The lack of pericardial stretch in constrictive pericarditis leads to increased ventricular coupling, characterized by septal flattening or inversion ("septal bounce") on early diastolic ventricular filling [48]. Because of the dissociation between intrathoracic and intracardiac pressure, this pattern is strongly influenced by respiration [2,70]. Septal abnormalities are most pronounced at onset of inspiration and rapidly fade away, while at onset of expiration an opposite (right-sided) septal shift occurs. CMR is appealing to functionally study patients with a clinical suspicion of constrictive pericarditis, and to visualize and quantify the degree of ventricular coupling. Giorgi and associates, using breath-hold cine CMR, reported early diastolic septal flattening or inversion in the majority of constrictive pericarditis patients, a pattern that was absent in restrictive cardiomyopathy patients [60]. Abnormalities were most evident in the basal septum, leading to a serpentine septal motion on cardiac long-axis view. This pattern was not present if the constrictive pericardium did not involve the right ventricle [60,61]. With the advent of real-time CMR, the effect of respiration on ventricular coupling could be dynamically exploited [18]. Francone et al. analyzed septal shape and position in patients with constrictive and inflammatory pericarditis and restrictive cardiomyopathy patients [72,73]. Constrictive pericarditis patients showed the typical respiratory pattern of septal abnormalities (Fig. 11) [see Additional file 6], while in restrictive cardiomyopathy a pattern similar to healthy volunteers was found. Quantification of the maximal respiratory septal excursion was helpful to differentiate both groups of patients. Also in patients with inflammatory pericarditis, increased septal excursion was found likely reflecting the decreased compliance of the inflamed pericardial layers. Real-time CMR may be appealing to depict increased ventricular coupling in patients with non or minimally thickened constrictive pericarditis where the morphologic pericardial abnormalities are minimal or absent.

Assessment of pericardial mobility is another potential application of dynamic CMR, and is appealing to differentiate normal from rigid pericardium (Fig. 8) [see Additional file 3]. The latter exhibits no or at most a limited displacement, while normal pericardium slightly moves during the cardiac cycle, often in synchronicity with the underlying myocardial motion. Moreover, the restricted expansion of the myocardium during cardiac filling abutting against the stiff pericardium can be well appreciated. These issues may be important in diagnosing patients with minimally or non-thickened constrictive pericarditis. CMR tagging may be of help to demonstrate extension of the fibrocalcific process into the underlying myocardium.

### Pericardial Masses

**Figure 12:**
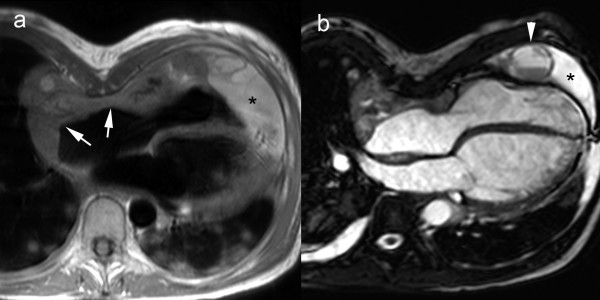
**Cardiac angiosarcoma**. **Axial T1-weighted spin-echo CMR (a), axial cine CMR (b)**. Mass arising from right atrial lateral wall (arrows) extending to anterior atrioventricular groove and pericardium. Presence of a pericardial effusion (*) with several nodular masses (arrowhead). Note the presence of multiple pulmonary metastases. Presence of pectus excavatum and right-sided pleural effusion.

Primary tumors of the pericardium are rare entities, occurring much less frequently than pericardial metastasis [74]. Pericardial mesothelioma is the most common primary malignant tumor of the pericardium, and is often associated with hemorrhagic pericardial effusion. Other primary tumors include malignant fibrosarcoma, angiosarcoma, and benign and malignant teratoma (Fig. 12) [see Additional file 7] [74-76]. CMR has the advantage to accurately delineate the tumor implantation and determine its relation to contiguous anatomical structures. Tissue characterization of pericardial tumors is often not possible, exceptions being lipoma and liposarcoma, which appear with high signal intensity due to their fatty content. Pericardial metastases are, in most cases, secondary to lung or breast carcinoma, leukemia, or lymphoma [77,78]. They are often associated with a large and hemorrhagic effusion that is disproportionate in size to the amount of tumor present. Since metastatic pericardial implants are often small, they might be difficult or even not visible on CMR.

Other entities that have a mass-like appearance are pericardial hematoma and pericardial gossypiboma. The appearance and CMR signal characteristics of a pericardial hematoma depends on the age of the collection [79]. Pericardial gossypiboma or foreign body granuloma should be considered in patients with previous cardiac surgery. In the pericardium, it includes surgical sponges in the pericardial space overlooked after pericardiectomy [11,79].

## Conclusion

The added value of CMR compared to the standard techniques used for assessment of patients with pericardial diseases has substantially increased in recent years, questioning whether this technique should not be considered the most appropriate non-invasive modality to study the pericardium. Strong points in favor of CMR are the integration of anatomic and functional information within a single examination, the ability for tissue characterization and to determine the presence and degree of inflammation and activity of disease, and the value of CMR to accurately assess the rest of the heart, in particular the myocardium, helpful in the differential diagnosis, which currently often remains a diagnostic challenge.

## Competing interests

The authors declare that they have no competing interests.

## Authors' contributions

Both authors (JB, MF) contributed in the design and writing of the manuscript, and approved the final manuscript.

## Appendix

### CMR strategies to evaluate the pericardium

* Pericardial morphology (*spin-echo CMR*/*cine CMR*)

- pericardial width/localization/extent

- pericardial delineation

* Pericardial layer/fluid characterization (*T1w/T2w spin-echo CMR*/*cine CMR*/*gadolinium-enhanced CMR*)

* Pericardial function

- motion pattern (*cine CMR*)

- fusion of pericardial layers (*CMR tagging*)

* Cardiac morphology (*spin-echo CMR, cine CMR*)

- size and shape of ventricles and ventricles

- myocardial morphology (restrictive cardiomyopathy)

* Cardiac systolic function (*cine CMR*)

- regional and global systolic ventricular function

* Cardiac filling (*velocity-encoded CMR*)

* Ventricular coupling (*real-time cine CMR*)

- ventricular septal shape and septal motion patterns

- respiratory-related septal shift

* Other findings (*spin-echo CMR/cine CMR/gadolinium-enhanced CMR*)

- myocardial enhancement (associated myocarditis? myocardial infiltrative or storage disease?)

- caval vein size

- pleural fluid/ascites

## Supplementary Material

Additional file 1 **Moderate pericardial effusion with signs of cardiac tamponade**. Moderate pericardial effusion with collapse of right atrial and right ventricular wall during the cardiac cycle. Click here for file

Additional file 2 **SPAMM (spatial modulation of magnetization) CMR tagging of the normal pericardium**. Pericardial tagging might be useful to evaluate fusion of pericardial layers. In normal circumstances, the continuity of the tags gets rapidly lost during cardiac contraction due to the shear motion between visceral and parietal layer. Click here for file

Additional file 3 **Calcified constrictive pericarditis**. The divergence in motion between the stiff, thickened pericardium and the normal contracting myocardium can be well appreciated on cine CMR. Click here for file

Additional file 4 **Inflammatory-constrictive form of constrictive pericarditis**. Real-time CMR in cardiac short-axis during free breathing shows increased ventricular coupling with inspiratory septal inversion and increased right-sided septal motion at onset of expiration. Click here for file

Additional file 5 **Focal constrictive pericarditis**. Cine CMR nicely shows the fixed appearance of the thickened pericardium (in contrast to the normal mobility of the non-thickened pericardium), focally impeding the expansion of right ventricle during cardiac filling. Click here for file

Additional file 6 **Increased ventricular coupling in constrictive pericarditis**. Dynamic imaging of the heart in cardiac short-axis during deep in- and expiration shows inspiratory septal inversion, and expiratory increased right-sided septal motion, leading to an increased respiratory septal shift. Click here for file

Additional file 7 **Cardiac angiosarcoma**. Dynamic MRI in axial plane shows mass arising from right atrial lateral wall extending to anterior atrioventricular groove and pericardium with presence of pericardial effusion and several nodular masses. Click here for file
